# Drama as a community engagement strategy for malaria in rural Cambodia

**DOI:** 10.12688/wellcomeopenres.12594.2

**Published:** 2018-05-16

**Authors:** Renly Lim, Rupam Tripura, Thomas J Peto, Ma Sareth, Nou Sanann, Chan Davoeung, Chea Nguon, Phaik Yeong Cheah

**Affiliations:** 1 Mahidol Oxford Tropical Medicine Research Unit, Faculty of Tropical Medicine, Mahidol University, Bangkok, Thailand; 2 Quality Use of Medicines and Pharmacy Research Centre, School of Pharmacy and Medical Sciences, University of South Australia, Adelaide, Australia; 3 Faculty of Medicine, University of Amsterdam, Amsterdam, Netherlands; 4 Nuffield Department of Clinical Medicine, University of Oxford, Oxford, UK; 5 Provincial Health Department, Battambang, Cambodia; 6 National Center for Parasitology, Entomology and Malaria Control, Phnom Penh, Cambodia; 7 The Ethox Centre, University of Oxford, Oxford, UK

**Keywords:** malaria, elimination, public engagement, community engagement, drama, Cambodia, evaluation

## Abstract

**Background**: Countries in Southeast Asia are working to eliminate multidrug-resistant falciparum malaria, a major cause of mortality in tropical regions. Malaria is declining but transmission persists in many rural areas and among forest workers and isolated populations. In these remote communities, conventional health services and education are limited. Mobilising and educating these populations require new approaches as many people are illiterate and do not attend village meetings. This article describes a qualitative study to assess the feasibility of a drama project as a community engagement strategy.

**Methods**: A drama project was conducted in twenty villages in Cambodia with three key messages: to use insecticide-treated bednets and repellents, to get early diagnosis and treatment, and to learn about risks of forest-acquired malaria. Qualitative interviews were conducted with the drama team members, village malaria workers, local health staffs and villagers, to explore the feasibility of using drama to engage the community and the associated challenges.

**Results**: 29 people were interviewed, which included 18 semi-structured interviews and one focus group discussion. Analysis of the interviews resulted in development of the following seven themes: i) exposure to malaria and engagement activities, ii) readiness and barriers to participation, iii) understanding and learning about malaria using drama, iv) entertainment value and engagement method preferences, v) challenges to community engagement, vi) future participation and vii) sustainability. The event saw a very positive response, with an encouraging average participation rate of 66%. The project faced several challenges including logistic problems, rescheduling due to raining season, and time- and budget-constraints.

**Conclusions**: Our evaluation demonstrated that the drama project was feasible in promoting awareness and understanding of malaria prevention and control. Audience members perceived drama as entertaining and as the preferred choice of engagement activity. Participatory drama could be considered as part of the community engagement for malaria elimination.

## Introduction

Countries in South East Asia are working towards the elimination of *Plasmodium falciparum*, a major cause of mortality in tropical regions ^1, 2^. In Cambodia, malaria prevention and control programmes have made significant progress and malaria cases have declined substantially ^3^. Unfortunately, emergent artemisinin-resistant *P. falciparum* threatens the recent gains ^4– 7^.

In January 2016, the Cambodian National Centre for Parasitology, Entomology and Malaria Control (CNM) announced the Malaria Elimination Action Framework (MEAF) 2016–2020, which aims to eliminate *P. falciparum* by 2020 and *P.vivax* by 2025 ^8^. The elimination efforts are underpinned by CNM’s village malaria worker (VMW) programme, where the VMWs provide early, free diagnosis and treatment in settlements with the highest prevalence ^9^. Achieving elimination is complicated by several factors. For example, in areas of low and falling transmission, those at highest risk of infection, forest goers and migrant groups, are the hardest to reach with health services and health education ^10, 11^. Moreover, the success of the VMW programme is predicated on the sustained motivation of isolated VMWs and local communities making use of the services they offer.

In rural communities affected by malaria, awareness and understanding of the disease and the VMW programme is indispensable to support elimination efforts. Such communities however often record lower literacy rates compared to urban areas: 77 percent compared to 93 percent in the 2014 census ^12^. Using printed media such as leaflets and posters to raise malaria awareness, may therefore be less successful in these areas. In developing countries, folk media such as folk songs, dramas, puppet shows and dance have been used as community engagement strategies for health education and to encourage research participation ^13– 15^. Khmer dramas which use comedy and music to tell stories with local references and language that resonates with villagers are popular in rural communities. In light of the need to convey messages about malaria and the VMW programme in a comprehensible and appropriate fashion, traditional Cambodian drama was used as a means to supplement existing text-based messaging on malaria and other efforts rolled out by the local authorities and education system. The Village Drama Against Malaria (VDAM) was a pilot project that sought to encourage people to prevent mosquito bites by using insecticide-treated nets and repellents, get early diagnosis and treatment from the VMWs, and to raise awareness about the risk of malaria in local forests. The aim of this qualitative study is to assess the feasibility of a drama project as a community engagement strategy to raise awareness of a health problem in the community and improve the community’s understanding of the disease in question, in this case malaria.

This paper describes:

i) the feasibility of the VDAM project as a community engagement strategy,
ii) the use drama and related workshops to convey the three key messages of the project (to use insecticide-treated nets and repellents, to get early diagnosis and treatment, and to raise awareness about the risk of malaria in local forests); and
iii) the challenges faced by the VDAM team, local team members and villagers.

## Methods

### Setting

The Village Drama Against Malaria (VDAM) project ^16^ was a joint initiative of the Battambang Provincial Health Department and the Mahidol-Oxford Tropical Medicine Research Unit (MORU), funded by the Wellcome Trust. MORU has a long-standing collaboration with the Battambang Provincial Health Department. The VDAM project was conducted alongside an on-going targeted malaria elimination study in six villages in Western Cambodia ^17^. Our ethical justification is that community engagement has the potential to strengthen the consent process by increasing community understanding about malaria, which in turn aids understanding of a malaria study by prospective research participants.

**Figure 1.**
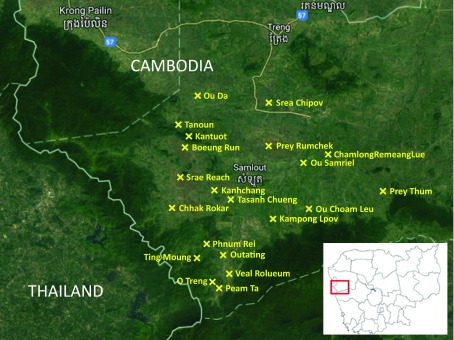
Location of the 20 drama villages in the Samlout district of Battambang province.

The project was conducted in 20 of the 49 remote villages in the Samlout District of Battambang Province, which is located in the western Cambodia near the Thailand border ( Figure 1). These 20 villages were selected by the provincial health department based on their high *P. falciparum* incidence from VMW records in 2014, and a proven prevalence of subclinical malaria infections from cross-sectional surveys in early 2015.

Village populations ranged from approximately 150 to 3000 residents with an average of 800 ( Table 1). Villagers are generally poor and most families are involved in small-scale agriculture. The villages typically have only basic infrastructure: poor access to potable water, sanitation and irrigation systems, limited electricity supplies, minimal post-primary education, and poor availability of health services. The villagers consist of majority ethnic Khmer with some ethnic minorities, chiefly the Por group, living in some villages close to the forests along the Thai border.

**Table 1.** Audience numbers, total population and percentage attendance in each of the twenty villages.

Number	Village	Audience numbers	Population	Percentage attendance
1	Outreng + Peamta	350	522	67.0
2	Vealroleum	250	291	85.9
3	Ting Moung	70	153	45.8
4	Phnom Rei	500	614	81.4
5	Ouda	650	1012	64.2
6	Srea Chipov	550	1537	35.8 ^#^
7	ChamlongRemeangLue	450	1450	31.0 ^#^
8	Tasanh Chueng	350	949	36.9
9	Boeung Run	600	814	73.7
10	Prey Thum/MaiKo	350	313	111.8 *
11	Ou Choam Leu	350	456	76.8
12	KampongLpov	450	298	151.0 *
13	Chhak Rokar	500	1781	28.1 ^#^
14	Srae Reach	450	562	80.1
15	Outating	150	164	91.5
16	Kanhchang	450	649	69.3
17	Prey Rumchek	600	3361	17.9 ^#^
18	Ou Samriel	550	793	69.4
19	Tanoun	450	822	54.7
20	Kantuot	550	1020	53.9

Key: ^#:^ These villages are very large in size, making it difficult for villagers to attend the drama event. The majority of villagers commute on foot. * : Percentage attendance exceeded 100% because participants came from nearby villages to attend the drama event.

### The VDAM community engagement project

The VDAM project combined Cambodian drama, art, music workshops and village concerts and competitions to provide health education in a fun and engaging way. Cambodian drama uses caricatures in a funny and irreverent manner and the VDAM productions did so to weave together stories and health education. The project mixed art and drama with modern technologies, such as using drones to film the villages, and social media ( Facebook https://www.facebook.com/dramaagainstmalaria and YouTube https://www.youtube.com/watch?v=ZD2oVgtaeM8) to share photos and videos across the province. The ‘Village Drama Against Malaria’ Facebook page was updated regularly after every drama performance depicting highlights of the 3-day events.

### Planning and involvement of local people

MORU has a long standing relationship with Cambodian health authorities. Since 2013, MORU has conducted malaria prevalence studies in villages in western Cambodia ^18^, and since 2015, targeted malaria elimination studies involving mass drug administration has been on-going in villages in Western Cambodia ^17^. All projects involve local stakeholders at the national, provincial and village level. In the VDAM project, local leaders and community are involved in its planning and implementation.

The VDAM team first introduced the project to and sought approval and support from the national authorities. Meetings were held in the target communities with village leaders, key opinion leaders identified by staff of the provincial health department and other prominent members to deliberate on the proposed project.

Professional drama performers were recruited in Battambang province. A series of workshops were organised to train the team on the malaria messages to incorporate into the drama performance. The final main script, storyline and key messages were approved by the provincial health authorities.

In each village, the team and village leaders met with the local community members and VMWs to explain the purpose of the project and to introduce the activities involved. Key opinion leaders in each village played an active role throughout the project development and progress.

### Drama workshops and performances

The project, which took place from June until September 2016, comprised a series of three-day workshops with the drama group travelling from one village to another every third day. During the workshops, villagers shared their talents and local stories which were integrated as part of the drama performance. School children were invited to participate to sing songs about malaria in Khmer and create drawings with the malaria theme. Teenagers and young people were invited to participate in the drama. On the third evening, the activities included singing competitions, songs and a quiz on malaria, followed by a 45-minute drama performance by both locals and professional drama team members. These events took place in the village square, usually near a school or temple, starting about 6pm and ending at 10pm or 11pm.

### Data collection

Our sampling method was purposive and limited by practicalities. In July 2016, 18 semi-structured interviews and one focus group discussion were conducted. The semi-structured interviews included six villagers who attended the drama performance, five professional actors, two VDAM organisers, two provincial health department staff, one VMW, one village leader and one local health staff. One focus group discussion took place, which involved eleven youths aged between 14 and 16 who participated in the performance.

Interviews were conducted by two interviewers (RL and PYC) in English and Khmer with the help of a Khmer social scientist (NS). All interviews, including the focus group discussion, were recorded with permission of the interviewees. In addition, data were obtained from field notes based on observations and informal conversations.

Interview guides explored the following areas:

i) personal history and experiences with malaria and community engagement,

ii) perceptions of malaria prevention and treatment,

iii) understanding of malaria messages conveyed via drama;

iv) opinions on the community engagement method,

v) challenges to organizing the community engagement or attending the event and

vi) feedback and suggestions on community engagement.

Audio recordings of interviews were transcribed and translated where necessary into English by professional translators. Two authors (RL and PYC) performed manual line-by-line coding of all interview transcripts in Microsoft Word. Using thematic analysis, the content of the codes were used to develop the themes that are presented below and to explore patterns of these themes across the transcripts/data sources.

### Ethical statement

The study was approved by the Oxford Tropical Research Ethics Committee (OXTREC; 1015-13), National Ethics Committee for Health Research Cambodia (18 ^th^ Feb 2016 NECHR 0051), and registered on clinicaltrials.gov (NCT01872702). Verbal informed consent was obtained from all participants prior to commencing the interviews and questionnaires. Verbal instead of written consent was obtained due to cultural reasons, and due to the low literacy level in the remote villages. The consent process was recorded using an audio recorder. Verbal consent for this study was approved by OXTREC in accordance with their guidelines on consent ( https://www.admin.ox.ac.uk/curec/resources/informed-consent/), as well as the local ethics committee. Approvals for the VDAM project, use of social media as well as filming and photography for all public events were obtained from the national and provincial authorities prior to the start of the project. 

## Results

Analysis of the data resulted in the following seven themes:

i) exposure to malaria and engagement activities,
ii) readiness and barriers to participation,
iii) understanding and learning about malaria using drama,
iv) entertainment value and engagement method preferences,
v) challenges to community engagement,
vi) future participation; and
vii) sustainability.

### Exposure to malaria and engagement activities

Interviews with local villagers revealed that either they themselves had contracted malaria, or know people who had contracted malaria. Villagers who had contracted malaria said that they suffered from its symptoms and economic complications, including inability to work and loss of income. Villagers who had personal experiences with malaria recognized its seriousness, and would seek diagnosis and treatment from VMWs. Some villagers shared stories of immigrants and outsiders who died after contracting malaria in the villages.

“Since one of the actors from this group died, then never has any other group which does the drama performance come to perform at this village any more.”
  (Village leader)

Community engagement activities were few and far between, and involved only showing videos and films, without active participation from the community. Staff from the local health authority felt that the existing health education methods, through healthcare centres, hospitals, advertisement (e.g. banners), media (e.g. television) and local service providers were limited and not comprehensive. Others commended the unique approach of the current participatory drama project.

### Readiness and barriers to participation

Many villagers expressed their readiness to participate in the drama activities, as reflected by an encouraging average participation rate of 66% (range 18% to 100%) ( Table 1). The number of attendees was between 70 and 650, with an average of 430 people. Many young people and children volunteered to participate during the workshops to showcase their performance talents. For many, this was the first time they received drama training and had the opportunity to perform in public.

Villagers said that their motivation to join the workshops stemmed from a desire to learn and understand more about malaria, and they hoped to get more information to eliminate malaria in their villages. Village leaders were interested in the project because they wanted to know more about the on-going projects conducted by MORU and the provincial health department.

The lack of transportation meant villagers often commuted on foot, making accessibility to the performance location potentially difficult. A few villages such as Srea Chipov and Prey Rumchek are very large in size. Villagers may not be able to attend the drama due to the distance, which potentially explains the lower coverage in these villages ( Table 1). Interviews with participants in other villages, however, showed that they had no problem walking to the pagodas or schools where the drama performances were conducted. The villagers who were interviewed said that they went with their entire families. They were happy to participate in the workshops, which they felt were 'real performances' instead of video shows and films.

### Understanding and learning about malaria using drama

To assess if the project can be used to convey the three key messages of the project (to use insecticide-treated nets and repellents, to get early diagnosis and treatment, and to raise awareness about the risk of malaria in local forests), interviewers asked the villagers about the lessons that they had learnt from the drama.

When asked about the drama, villagers faced no difficulty recapitulating the storyline, which comprised two main characters, one of whom slept under a bednet while the other slept without protection. The latter contracted malaria and went to meet the VMW, who performed a blood test to confirm the malaria diagnosis. He was given a three-day malaria medication and later recovered. The respondents felt that the team gave simple and straightforward key messages which were easy to understand to the local community, including people who were illiterate.

Some villagers said that the drama would bring about behavioural changes in their daily lives. Compared with before the drama, solely relying on mosquito nets, they would now use mosquito repellents during the day. Men who go to the forest would bring mosquito nets with them and wear long-sleeved clothing.

“.......Now they (the husbands) use repellent. For the men when they go to the forest, they bring the mosquito net there and wear the long trousers and long shirt.......”(audience)

### Entertainment value and engagement method preferences

The drama performance was described as funny and entertaining, and made learning and understanding about malaria much more interesting compared with the conventional health education methods. Villagers especially enjoyed watching children dressed as mosquitoes while singing songs about malaria. A quote from the children’s song:

“Everybody needs to know malaria;
The mosquitoes they come biting;
Stay inside mosquito nets,
Everybody needs to know malaria;
Prevent mosquitoes from hunting;
Stay alive, do not miss the mosquito net.”

Villagers were seen dancing away on the open ground in front of the stage. The catchy tunes meant villagers could easily join in the singing, and often were able to remember the messages in the songs.

Amongst the activities held during the evening (singing competitions, songs and a quiz on malaria, drama performance), drama was reported to be the favourite, followed by songs on malaria. Audience members used Facebook, which is very popular with Cambodians, to upload pictures and videos of the performances.

“Actually I liked most of the program, however one section that I am interested in was the drama because the indigenous people find it easy to understand. The short drama like people sleeping inside the mosquito net and sleeping outside the mosquito net (referring to drama plot).”
(audience)

The provincial health department and local healthcare staff commented that health education without entertainment may not be appealing to the villagers. Combining arts and science led people to remember key messages better. Using drama to attract people in the village, and incorporate key messages about malaria achieved both entertainment and education purposes.

### Challenges to community engagement

Reaching the forest goers and migrant population was a particular challenge. On each visit, forest workers typically spend one week to ten days in the forest; they make two trips per month, whereas migrants move in and out of the villages depending on planting and harvest seasons. For many forest workers who were unable to attend the event, their partners said that they would get their husbands to use repellents when they go into the forest in the future.

The village leader and local health staff raised the villagers' concerns regarding rumours that healthcare personnel came to the villages to collect their blood and sell it. Some respondents also described how other villagers believe in spiritual and psychic mediums to cure their illnesses. Some villagers reported limited understanding of the project aim, and this led to reservations about participation. Questions raised included, 'who are [the drama team]?', 'where do they come from?' and 'what are they doing here?'.

“…….because they believe in the superstitions like they believe in burning incense for the offering. They said they don’t want to do blood test or to have vaccinations.”
  (audience)
“And some people they said that oh...maybe take their blood for selling. Because they don't know. Because in Cambodia they sell blood.”
  (local health staff)

Social relationships in Cambodia embody a hierarchical pattern. The villagers' attitudes and participation appear to be heavily influenced by the village leaders and monks, who are respected members of the community. Establishing trust and good relationships with local village and spiritual leaders, and gaining their support implied subsequent participation from the villagers. Our project which incorporated use of a community-directed approach while maintaining a close collaboration between MORU staffs and local leaders contributed to the success of this project.

“They followed the head of the indigenous community because normally the person who is the head of the indigenous people has influence and the indigenous people listen to the head.”
(audience)
“…….for the program to run smoothly depends on the local people and stakeholders joining to help each other like all of you come to help my community. ……. Unless the people who are responsible in the village cooperate and work together the work will not run smoothly.”
(audience)

The VDAM organisers commented that the project which was conducted from June to September 2016 coincided with the rainy season in Cambodia. Roads that were already a bumpy ride for motorists were further battered due to the heavy downpour, making them inaccessible via small trucks used by the local drama team. The performance schedule often needed rearrangements because bridges connecting villages collapsed due to the flood.

### Future participation

Many interviewees requested that the VDAM team organise the event again and said that they would participate in the future because very few events are organised in their villages. Some wanted their family members to participate in the event so that they would remember the key messages more effectively. The local health staff commented health education without properly engaging the community results in minimal impact.

“I think it would be great for you to come many times because sometimes when we just use our words to teach people it doesn’t really directly impact them.”
(audience)
“If possible, my suggestion is to have this event every year because if we go for a long time without doing the events then the community could forget it but if we do it every year or two times a year then it will help to remind the community about using the mosquito nets whenever they sleep at home or out in the forest or farming. Then they can apply the message because they have seen the drama every year.”
(audience)

### Sustainability

The MORU team worked closely with the provincial health department and village leaders. Interviews with these key members suggested that villagers gained a better understanding of the purpose of malaria elimination projects after they conveyed these messages to the villagers. Some villagers said they were now more willing to do blood testing, which was conducted as part of malaria research conducted by MORU.

Interviewee: “For me, I really think this is very valuable for the community.”
Interviewer: “Really, uncle? So it provides benefits for the community?”
Interviewee: “Yes, it really does. And recently people have involved in activities, for example: blood testing and so on.”
  (audience)

During the focus group discussion, youths felt that they learned how to perform in public and to express their thoughts. They further requested for materials related to ways of preventing malaria so that they could spread such awareness in schools. At the end of the project, video recordings and photos of the workshops and performances in the form of DVDs were distributed to all village leaders as souvenirs. All photos and videos were taken at public places and with formal written permission from the national and provincial authorities.

## Discussion

Our results showed that the VDAM project was a feasible method to engage the rural communities and to convey the key malaria messages. Villagers said they learned more about malaria through the drama, enjoyed watching and participating in the performances and were keen to participate in future events. To our knowledge, the current paper is the first assessment of using drama to engage the community for malaria control and elimination.

### Drama within a community engagement strategy

Community engagement is increasing recognized as integral to successful and ethical health-related research ^19^. To maximize the efficiency of the community engagement programme, a combination of 'top-down' and 'bottom-up' approaches are needed ^20^. Such a combination of approaches was taken to promote VDAM project. Taking 'top-down' approach, support was garnered from the provincial health department, village leaders and other respected members of the community which would then convince the local communities to participate. Taking a 'bottom-up' approach, local communities were encouraged to actively participate in the planning and execution of the 3-day event and to contribute stories and ideas to the drama performance, an approach the villagers claimed they have never experienced before.

Our community engagement project used traditional Cambodian drama, a visual method of engagement, to incorporate key messages about malaria prevention and elimination. Our community engagement project used traditional Cambodian drama, a visual method of engagement, to incorporate key messages about malaria prevention and elimination. Participatory visual methods as a community engagement strategy can increase participant engagement and communication, allow participants to share their stories, opinions and experiences, and foster relationships between researchers and the participants ^21, 22^. A similar method was used in a study in Laos which utilized traditional folk songs known as 'lam' as an educational medium for preventing HIV/AIDS ^13^. Based on interviews in the focus group discussions, the authors concluded that traditional folk songs could be used to motivate individual behavioural changes and to encourage community action for disease prevention. However, traditional songs were less enticing to the younger generations who preferred pop songs. Our engagement project used drama and various music genres which appealed to both the younger and older generations.

### Challenges to drama as community engagement

Due to the timing of approvals, the project was conducted during the rainy season. Travelling from one village to another was considerably slower and everything took longer. Performance dates were often rescheduled due to the degraded road conditions. Frequent heavy downpours also made it more difficult for villagers to participate in the drama workshops and performance. Malaria incidence occurs throughout the year but most malaria cases are confined to the months during or directly after the rainy season; it would have been more appropriate to deliver the malaria messages before the rainy season begins. In a few villages with large population of over 1000 people, the villages consisted of several hamlets which were scattered. It was too far for villagers to walk to the event location, which resulted in lower participation rates in those villages.

Another challenge faced by the VDAM team was the time pressure. It was crucial to allocate enough time in each village without compromising the ability to convey the key malaria messages. However, with twenty villages to perform in, the length of time spent in each village was limited to only three days. As a result, we were unable to reach a large number of young men who were mainly forest workers who spend a significant amount of time in the forests, and the mobile and migrant population who were not in the villages.

### Limitations

Since this engagement project had time and budget limitations, a longitudinal evaluation was not feasible. Furthermore, it was not possible to differentiate the VDAM project from other engagement and educational projects provided by the provincial health department, schools and VMWs. Since there was no before and after comparison made, it was not possible to determine the extent of changes in knowledge of malaria amongst villagers. The ultimate indicator of success is the elimination of malaria in these villages, which would be attributable to many factors.

Our sample size was small as we were limited by practicalities: chiefly the interviewers could not attend all of the remote villages where the drama took place. The performances tended to end quite late at night and villagers had to go home immediately after the event ended to wake up early the next day. Many people also appeared to be shy and somewhat reluctant to be interviewed. The small sample size also resulted in the large overlap between the areas explored in the interview guides and the resulting themes.

## Conclusions

We conclude that although there were many challenges, village drama and its associated activities such as competitions and workshops is feasible as a community engagement strategy. Audience members recalled the plot of the performance and the malaria-related messages, indicating that the key messages were clear and concrete. Villagers perceived drama as entertaining and as the preferred choice of community engagement activity. Using drama is a promising way to engage communities and could be considered as part of the community engagement for malaria elimination.

## Abbreviations

CNM: Cambodian National Centre for Parasitology, Entomology and Malaria Control; MEAF: Malaria Elimination Action Framework; MORU: Mahidol-Oxford Tropical Medicine Research Unit; VDAM: Village Drama Against Malaria; VMW: village malaria worker.

## Ethical statement

The study was approved by the Oxford Tropical Research Ethics Committee (OXTREC; 1015-13), National Ethics Committee for Health Research Cambodia (18 ^th^ Feb 2016 NECHR 0051), and registered on clinicaltrials.gov (NCT01872702). Verbal informed consent was obtained from all participants prior to commencing the interviews and questionnaires. Verbal consent for this study was approved by OXTREC in accordance with their guidelines on consent ( https://www.admin.ox.ac.uk/curec/resources/informed-consent/) as well as the local ethics committee. Approvals for the VDAM project, use of social media as well as filming and photography for all public events were obtained from the national and provincial authorities prior to the start of the project.

## Data availability

Due to ethical and security considerations, the data that supports the findings in this study can be accessed only through the Data Access Committee at Mahidol Oxford Tropical Medicine Research Unit (MORU). The data sharing policy can be found here: http://www.tropmedres.ac/data-sharing. The application form for datasets under the custodianship of MORU Tropical Network can be found as a Supplementary File.

## Supplementary material

Supplementary File 1: The application form for datasets supporting this research, under the custodianship of MORU Tropical Network.
