# Supplementary material for: Drama as a community engagement strategy for malaria in rural Cambodia
Source: Wellcome Open Res. 2018 May 16;2:95. Originally published 2017 Sep 29. [Version 2] doi: 10.12688/wellcomeopenres.12594.2 (PMC5645711; doi:10.12688/wellcomeopenres.12594.2)
Supplement: Peer review history

# Peer review: Referee response for version 2

**DOI:** 10.21956/wellcomeopenres.15916.r33154

**Published:** 2018-06-04

**Authors:** Gillian F. Black

**Affiliations:** 1 Sustainable Livelihoods Foundation, Cape Town, South Africa

Once again I would like to congratulate Renly Lim and co-authors on writing an important article that highlights how the creative arts and a participatory approach can contribute meaningfully to enhanced community awareness about the major public health risk of malaria. 

 The changes that have been made to the manuscript in response to reviewer 3 have certainly helped to improve the paper.

 There has been no written reply to my previous comments. I have read through the manuscript again and recognized that there have been no responses to my suggestions, apart from one comment that referred to acknowledging a limitation of the study with respect to the thematic development.  

 I would very much like to see this manuscript fully approved as soon as possible. There are however certain areas of the paper that need to be amended beforehand. 

 Please address the following:   

 The authors explain that the project took place between June and September 2016, and that the interviews and focus group discussions were conducted in July of that year. Details are provided about the groups who were included in these processes, and the number of respondents. Were all of the respondents from the same village? (i.e. are all responses based on a single 3-day workshop event?). It is important to know this as it will indicate which of the study areas are represented in the results and conclusions. (At several points in the paper, the authors refer to ‘the’ event or ‘the’ performance, which indicates that the results are indeed based on the implementation of a single workshop activity. Please do clarify if this was the case. 

 In the discussion, the authors mention that to their knowledge ‘the current paper is the first assessment of using drama to engage ‘the community’ for malaria control and elimination’. The work of Aitkinson and Vallely is cited with reference to the need for both bottom up and top down approaches in community engagement strategies. However, these authors have also published multiple papers on the use of drama for malaria-related CE in Vanuatu which should be more clearly acknowledged in the references. 

 The groups that participated in the interviews/focus group discussions, and the number of respondents in each of these groups, is described on page 5. For example, we know that there was 1 local health staff respondent and 1 village leader respondents. However, at several points in the results sections, the writing indicates that there were multiple respondents in these groups. (‘Staff form the local health authority felt…’, and ‘ Village leaders were interested because…’. The text needs to more accurately reflect the singular nature of these responses.****

 I feel that sustainability is not a relevant theme to be discussed this paper, based on the results of the data analysis that are described. The actual responses cited in this section refer to better understanding about malaria elimination projects that were gained through the engagement approach, which would seem to fit under theme 3 (understanding and learning about malaria using drama). Also, increased willingness of villagers to do blood testing does not speak to the sustainability of the engagement approach that is being evaluated, but rather to its impact. Revisit this section and think about which parts can moved to other themes, and which parts can be moved to the methods or discussion sections.     

 In addition, the ethical statement on page 5 is still duplicated on page 8. Please remove one of these sections.

I have read this submission. I believe that I have an appropriate level of expertise to confirm that it is of an acceptable scientific standard, however I have significant reservations, as outlined above.

# Peer review: Referee response for version 2

**DOI:** 10.21956/wellcomeopenres.15916.r33153

**Published:** 2018-05-23

**Authors:** Alun Davies

**Affiliations:** 1 Kenya Medical Research Institute-Wellcome Trust Research Programme, Kilifi, Kenya; 2 The Centre for Tropical Medicine, University of Oxford, Oxford, UK

The authors have satisfactorily addressed my comments. This is a very interesting article.

I have read this submission. I believe that I have an appropriate level of expertise to confirm that it is of an acceptable scientific standard.

# Peer review: Referee response for version 1

**DOI:** 10.21956/wellcomeopenres.13638.r26533

**Published:** 2017-10-13

**Authors:** Alun Davies

**Affiliations:** 1 Kenya Medical Research Institute-Wellcome Trust Research Programme, Kilifi, Kenya; 2 The Centre for Tropical Medicine, University of Oxford, Oxford, UK

Overall, I feel that the novel approach described in this project is worthy of disseminating to wider engagement audiences. The study is described in a fairly clear way; however, aspects could be improved on to strengthen the article.

 Firstly, I would encourage the authors to clearly reference the Facebook and YouTube links explicitly with hyperlinks within the document if this is possible. The footage and images within these links clearly convey: the nature of the engagement; the participants and audiences involved in the activities; some of the challenges the authors and implementers encountered; and the sheer enjoyment of the participants in taking part in the activities. These are incredibly beautiful and striking images which bring the article alive and so are worth making the most of (assuming of course that the participants are happy for their images to be shared).

 There is a risk that readers may interpret the project as using a ‘deficit model’ of engagement, where educated researchers enter ‘illiterate’ communities to fill the knowledge gaps. Though the main aim of the activities described is explicitly stated as addressing health knowledge gaps related to Malaria control, I feel that more of an emphasis could be made of the close collaboration between researchers and community members required in implementing this project, drawing on existing local talent and knowledge, towards addressing goals of engagement/health promotion. This collaboration was especially evident in the videos. In relation to this, if the data-set included evidence of a strengthening relationship, mutual-understanding or trust between researchers and participants, then this would be worth including.

 There is some ambiguity about the overall aim of the engagement/research. The abstract states that the purpose of the project is to assess the feasibility of a drama project as a community engagement strategy. The description however, lacks an ethical argument as to why the authors selected health-promotion activities as a vehicle for engaging communities. Playing devil’s advocate, it could be argued that health promoting activities such as “using insecticide-treated bednets and repellents, etc.” rather than being the role of health researchers, is the work of the the Cambodian National Centre for Parasitology, Entomology and Malaria Control (CNM), through their Malaria Elimination Action Framework (MEAF) 2016–2020. Since the discussion presents a case that this is an engagement project ‘integral to successful and ethical health-related research,’ more clarity is needed in arguing why conducting health-promotion activities addresses ethical goals of community engagement. For example, did the implementers feel that they were well placed to conduct these activities, given their knowledge of malaria, to conduct health promotion? Was it a form of addressing ‘justice’ and ‘beneficence’ in research through offering community members means of improving their health? Was the purpose of the engagement to strengthen community members understanding of Malaria control, in order to complement informed consent for a specific MORU study (addressing ‘respect for persons’)? Or was it a means of strengthening relationships between research host communities and researchers (there is some evidence of this – leaders felt that participation offered an opportunity to learn about the work of MORU)? … or was it a combination of all of these? I feel that using health-promotion as a means of engagement is entirely justifiable (see for example Participants in the CE and Consent Workshop (2013) ^1^ or (Nakibinge et al., 2009 ^2^), but I think the ambiguity could be cleared with a short description/justification of why they are using it.

 In relation to the above paragraph it would be helpful to provide a clearer description of the Malaria Elimination Action Framework (MEAF) 2016–2020. Is this a guideline document, or a group of government interventions, or both? Is the Battambang Provincial Health Department responsible for implementing this framework? If so, what are they already doing on the ground? Does MORU have a “Malaria elimination’, or related study which this engagement project is part of, or is the project a stand-alone?

 I agree with other referees with regards to the following points

- Requiring reference to the value of working with participatory visual methods as a community engagement approach in public health and health research
- The relatively small sample size, but that this is acknowledged as a limitation
- The overlaps with regards to the data presented within the findings themes and how these could be streamlined

In addition to the above points, I would have liked to have seen more participant quotes to highlight some of the qualitative findings, for example, in the following areas: a) the drama bringing about reported behaviour change; b) a quote from the children’s song; c) a quote highlighting that drama was the preferred activity; d) evidence of establishing trust by community leaders translating to subsequent participation; and e) evidence of a desire for further participation.

 This article provides a description of the creative use of a culturally appropriate method to engage communities with health and health research. As such it is worthy of being indexed.

I have read this submission. I believe that I have an appropriate level of expertise to confirm that it is of an acceptable scientific standard, however I have significant reservations, as outlined above.

## References

[1] Participants in the Community Engagement and Consent Workshop , Kilifi, Kenya , March 2011: Consent and community engagement in diverse research contexts. *J Empir Res Hum Res Ethics*.2013;8(4) : 10.1525/jer.2013.8.4.1 1-18 10.1525/jer.2013.8.4.1 24169417PMC4836561

[2] NakibingeSMaherDKatendeJKamaliAGrosskurthHSeeleyJ: Community engagement in health research: two decades of experience from a research project on HIV in rural Uganda. *Trop Med Int Health*.2009;14(2) : 10.1111/j.1365-3156.2008.02207.x 190-5 10.1111/j.1365-3156.2008.02207.x 19207175

# Response

**Published:** 2018-05-13

**Authors:** Renly Lim

**Affiliations:** Mahidol Oxford Tropical Medicine Research Unit, Thailand

Dear Dr Davies,

We would like to thank you for taking the time to review our paper and for the helpful suggestions. Below is our point-by-point response:

**Comment 1:** Firstly, I would encourage the authors to clearly reference the Facebook and YouTube links explicitly with hyperlinks within the document if this is possible. The footage and images within these links clearly convey: the nature of the engagement; the participants and audiences involved in the activities; some of the challenges the authors and implementers encountered; and the sheer enjoyment of the participants in taking part in the activities. These are incredibly beautiful and striking images which bring the article alive and so are worth making the most of (assuming of course that the participants are happy for their images to be shared).

Response 1: We agree with the reviewer's suggestion and have added the Facebook and YouTube links in the methods, 'The VDAM community engagement project' section. We obtained formal written permission from the national and provincial authorities as well as verbal consent prior to taking any images and video footages, and have obtained permission to share these materials.

**Comment 2: **There is a risk that readers may interpret the project as using a ‘deficit model’ of engagement, where educated researchers enter ‘illiterate’ communities to fill the knowledge gaps. Though the main aim of the activities described is explicitly stated as addressing health knowledge gaps related to Malaria control, I feel that more of an emphasis could be made of the close collaboration between researchers and community members required in implementing this project, drawing on existing local talent and knowledge, towards addressing goals of engagement/health promotion. This collaboration was especially evident in the videos. In relation to this, if the data-set included evidence of a strengthening relationship, mutual-understanding or trust between researchers and participants, then this would be worth including.

Response 2: Agree with the reviewer's comments that we should elaborate on the close relationship between our staff and the local community. We have added the following in the methods and results section:

-Methods

*Planning and involvement of local people*

MORU has a long standing relationship with Cambodian health authorities. Since 2013, MORU has conducted malaria prevalence studies in villages in western Cambodia [18], and since 2015, targeted malaria elimination studies involving mass drug administration has been on-going in villages in Western Cambodia. All projects involve local stakeholders at the national, provincial and village level.  In the VDAM project, local leaders and community  are involved in its planning and implementation.

-Results

Our project which incorporated use of a community-directed approach while maintaining a close collaboration between MORU staffs and local leaders contributed to the success of this project.

"…….for the program to run smoothly depends on the local people and stakeholders joining to help each other like all of you come to help my community. ……. Unless the people who are responsible in the village cooperate and work together the work will not run smoothly."

(audience)

**Comment 3:** There is some ambiguity about the overall aim of the engagement/research. The abstract states that the purpose of the project is to assess the feasibility of a drama project as a community engagement strategy. The description however, lacks an ethical argument as to why the authors selected health-promotion activities as a vehicle for engaging communities. Playing devil’s advocate, it could be argued that health promoting activities such as “using insecticide-treated bednets and repellents, etc.” rather than being the role of health researchers, is the work of the the Cambodian National Centre for Parasitology, Entomology and Malaria Control (CNM), through their Malaria Elimination Action Framework (MEAF) 2016–2020. Since the discussion presents a case that this is an engagement project ‘integral to successful and ethical health-related research,’ more clarity is needed in arguing why conducting health-promotion activities addresses ethical goals of community engagement. For example, did the implementers feel that they were well placed to conduct these activities, given their knowledge of malaria, to conduct health promotion? Was it a form of addressing ‘justice’ and ‘beneficence’ in research through offering community members means of improving their health? Was the purpose of the engagement to strengthen community members understanding of Malaria control, in order to complement informed consent for a specific MORU study (addressing ‘respect for persons’)? Or was it a means of strengthening relationships between research host communities and researchers (there is some evidence of this – leaders felt that participation offered an opportunity to learn about the work of MORU)? … or was it a combination of all of these? I feel that using health-promotion as a means of engagement is entirely justifiable (see for example Participants in the CE and Consent Workshop (2013) ^1^ or (Nakibinge et al., 2009 ^2^), but I think the ambiguity could be cleared with a short description/justification of why they are using it.

Response 3:

We agree that the aim has not been stated clearly. We have added the following in the introduction and methods section:

-Introduction

The aim of this qualitative study is to assess the feasibility of a drama project as a community engagement strategy to raise awareness of a health problem in the community and improve the community’s understanding of the disease in question, in this case malaria.

-Methods

The VDAM project was conducted alongside an on-going targeted malaria elimination study in six villages in Western Cambodia [17]. Our ethical justification is that community engagement has the potential to strengthen the consent process by increasing community understanding about malaria, which in turn aids understanding of a malaria study by prospective research participants.

**Comment 4: **In relation to the above paragraph it would be helpful to provide a clearer description of the Malaria Elimination Action Framework (MEAF) 2016–2020. Is this a guideline document, or a group of government interventions, or both? Is the Battambang Provincial Health Department responsible for implementing this framework? If so, what are they already doing on the ground? Does MORU have a “Malaria elimination’, or related study which this engagement project is part of, or is the project a stand-alone?

Response 4: The Malaria Elimination Action Framework (MEAF) 2016–2020 is a national guideline delineating five strategic plans which would be implemented by 2020 in the hope of eliminating malaria in Cambodia. The framework is currently being implemented nationally by the Cambodian National Centre for Parasitology, Entomology and Malaria Control (CNM) and all implementing partners including the Battambang Provincial Health Department.

The community engagement project described in our paper was conducted alongside an on-going targeted malaria elimination study providing mass drug administration in six villages in Western Cambodia. We have clarified this in the methods section.

**Comment 5:** I agree with other referees with regards to the following points:

- Requiring reference to the value of working with participatory visual methods as a community engagement approach in public health and health research
- The relatively small sample size, but that this is acknowledged as a limitation
- The overlaps with regards to the data presented within the findings themes and how these could be streamlined

Response 5:

We agree with the reviewer's suggestions. We have added two references discussing the value of participatory visual methods as a community engagement in the discussion section.

-Discussion

Our community engagement project used traditional Cambodian drama, a visual method of engagement, to incorporate key messages about malaria prevention and elimination. Participatory visual methods as a community engagement strategy can increase participant engagement and communication, allow participants to share their stories, opinions and experiences, and foster relationships between researchers and the participants [21,22].

With regards to the small sample size, this was mainly due to time constraint and language barrier. We were only able to arrange a limited number of short visits from MORU in Thailand where members of our evaluation group were working at, to the remote villages which were 10 to 12 hours away on road. In addition, having only one local translator who also held other responsibilities within the project, we could only conduct a few interviews during each visit. Hiring a translator from other places was not an option because we needed someone who has established rapport with the locals, in order to gain trust and permission to conduct interviews.

We are aware that there are overlaps between the areas explored in our interview guides and the resulting themes. Given the small sample size, the data that we were able to extract were also limited. As a result, the responses did not vary. We have added this to our study limitation.

**Comment 6:** In addition to the above points, I would have liked to have seen more participant quotes to highlight some of the qualitative findings, for example, in the following areas: a) the drama bringing about reported behaviour change; b) a quote from the children’s song; c) a quote highlighting that drama was the preferred activity; d) evidence of establishing trust by community leaders translating to subsequent participation; and e) evidence of a desire for further participation.

Response 6:

We agree and have added more quotes from the interviews. The following quotes have been added in the results section:

i) on behavioural changes;

".......Now they (the husbands) use repellent. For the men when they go to the forest, they bring the mosquito net there and wear the long trousers and long shirt......."

(audience)

ii) quote from the children’s song;

"Everybody needs to know malaria;

The mosquitoes they come biting;  

Stay inside mosquito nets,

Everybody needs to know malaria;

Prevent mosquitoes from hunting;

Stay alive, do not miss the mosquito net."

iii) a quote highlighting that drama was the preferred activity;

"Actually I liked most of the program, however one section that I am interested in was the drama because the indigenous people find it easy to understand. The short drama like people sleeping inside the mosquito net and sleeping outside the mosquito net (referring to the drama plot)."

            (audience)

d) evidence of establishing trust by community leaders translating to subsequent participation;

"They followed the head of the indigenous community because normally the person who is the head of the indigenous people has  influence and the indigenous people listen to the head."

(audience)

e) evidence of a desire for further participation.

"I think it would be great for you to come many times because sometimes when we just use our words to teach people it doesn’t really directly impact them."

(audience)

"If possible, my suggestion ​is to have this event every year because if we go for a long time without doing the events then the community could forget it but if we do it every year or two times a year then it will help to remind the community about using the mosquito nets whenever they sleep at home or out in the forest or farming. Then they can apply the message because they have seen the drama every year."

(audience)

# Peer review: Referee response for version 1

**DOI:** 10.21956/wellcomeopenres.13638.r26534

**Published:** 2017-10-06

**Authors:** Mary Chambers

**Affiliations:** 1 Oxford University Clinical Research Unit (OUCRU), Ho Chi Minh City, Vietnam

It is timely to have a publication describing the strengths and weaknesses of  community engagement activities. Pressure from funders and increasing researcher interest in engagement means that more biomedical trials and research studies have a community engagement component. However, publications evaluating their effectiveness are scarce.

 The authors clearly state the reasons for and the methods of engagement. The genre of engagement seems appropriate for the population served, as backed by the evaluation. The data collection is described clearly, although I would like the authors to clarify the timeframe between the activities and the interviews/focus group. This is relevant if they are assessing recall of the theatre script content and attributing the engaging methods of health promotion for people's ability to remember the key health messages.

 The number of participants is small and ideally they would have interviewed more villagers, and at different time points. The authors acknowledge this as a limitation to the study.

 The discussion is clear and conclusions appropriate. However, I would question the overall conclusion that village drama could be considered as part of CE for malaria elimination without some discussion as to whether these activities are prohibitively costly for local health systems to conduct. The question of sustainability as an imbedded CE/public health activity is not addressed. Perhaps the conclusion that they may be effective as part of an externally funded research engagement strategy would be more appropriate.

 These minor limitations do not prevent this from being a well written and useful contribution to the literature about community engagement in a biomedical research setting.

I have read this submission. I believe that I have an appropriate level of expertise to confirm that it is of an acceptable scientific standard.

# Peer review: Referee response for version 1

**DOI:** 10.21956/wellcomeopenres.13638.r26538

**Published:** 2017-10-05

**Authors:** Gillian F. Black

**Affiliations:** 1 Sustainable Livelihoods Foundation, Cape Town, South Africa

**Is the work clearly and accurately presented?**

 For the most part, the work is clearly and accurately presented, but important clarifications are required at certain points.

 It is emphasized throughout that drama is the key engagement strategy being evaluated. However, the VDAM community engagement approach (at each site) comprised of a 3-day workshop including art, music, village concerts and competitions, with a (participatory) drama performance taking place on the final evening. As far as I can understand, drama was not used in isolation in any of the participant villages. So using the term 'drama project' is questionable. The authors could perhaps consider another term that better reflects the multi-pronged nature of the community engagement approach taken.  

 The authors explain that the project took place between June and September 2016, and that the interviews and focus group discussions were conducted in July of that year. Details are provided about the groups who were included in these processes, and the number of respondents. Were all of the respondents from the same village? (i.e. are all responses based on a single 3-day workshop event?). It is important to know this as it will indicate which of the study areas are represented in the results and conclusions. (At several points in the paper, the authors refer to ‘the’ event or ‘the’ performance, which indicates that the results are indeed based on the implementation of a single workshop activity. Please do clarify if this was the case).

 It is explained that the project also included modern technologies such as drones to film the villages and social media to share photos and videos across the province. These elements of the engagement process do not inform the research findings described in the article. I was left wondering why this information was included in the paper, what the main purpose of this province wide dissemination was, and what the ethical implications of these disseminations approaches were.

 The average rate of event participation is calculated to be 66% across all the villages, and this statistic is highlighted in the paper abstract. The authors explain that a single 3-day workshop was held for each village, that there is appreciable variation in the size of the villages, and that the majority of villagers commute on foot, making it potentially difficult for residents of the largest villages to attend the drama events (due to walking distance). The suggestion that walking distance may explain lower participation rates in larger villages could be substantiated by a formal but simple analysis of population size versus percent attendance. A comment on the feasibility/practicality/value of implementing more than one workshop in the larger villages would be helpful, too. 

 I felt that certain parts of the results section (e.g. page 6 ‘Combining arts and science…’ and ‘Social relationships in Cambodia…’) would be more appropriately placed in the discussion.   

**Current literature**

 In the discussion, the authors mention that - to their knowledge - ‘the current paper is the first assessment of using drama to engage ‘ the community’ for malaria control and elimination’. M Frishkopf and his colleagues have recently published research showing that a performing arts public health intervention in northern Ghana was effective to convey key messages about malaria transmission to local audiences (Frishkopf M. et al, 2016 ^1^)

 Additional reference to growing international recognition of both the value and ethics of community engagement in health-related research could be included. (King KF et al., 2014 ^2^. Wallerstein and Duran, 2010 ^3^). 

 The key community engagement intervention strategy that is discussed in this paper is participatory drama. Drama is a visual method of engagement. Some reference to the value of working with participatory visual methods as a community engagement approach in public health and health research would add an important element to the current article (Mitchell and Sommer, 2016 ^4^)

**Are sufficient details of methods and analysis provided to allow replication by others?**

 On page 5, the authors explain that their sampling method was purposive and limited by practicalities, but. To enable replicability, it would have been helpful to be given a brief description of the process through which interview respondents and focus group members were selected.   

 The authors also explain that semi-structured interviews were done with various groups, including five professional actors one village malaria worker. The results section describes thematic key responses from different participant groups – especially the villagers - but there is no mention of responses from the actors or the VMWs. A complete understanding of the feasibility of the engagement strategy will only be gained through including the perspectives of all research respondents in the analysis.     

 The description of the drama workshops and performances indicates that the drama was partly developed before the village engagement activities began, and then further developed during the workshop. This further development included participation from teenagers and young people, at each site, by the incorporation of their personal stories and experiences. This process of drama development is a very important part of the methodology and approach to community engagement. I would suggest that a bit more detail is provided here as to the steps taken, for the purposes of replicability by others in the field. In the interest of the word count, this could perhaps be written in detail as supplementary material.

 The section describing the main storyline for the play is currently included in the results section (page 6). It does not describe the results of the data analysis, and would be more appropriately placed in the methods section. However, the storyline description is not necessary to the understanding of the project and could be left out entirely.  

**Are the conclusions drawn adequately and supported by the results?**

 Seven themes were surfaced through analysis of the data generated by the interviews and focus group discussions. There is a large overlap between the areas explored in the interview guides and the resulting themes. This is not entirely surprising, given the small sample size in each respondent group, but it is worth noting as a study limitation.  

 The groups that participated in the interviews/focus group discussions, and the number of respondents in each of these groups, is described on page 5. For example, we know that there was 1 local health staff respondent and 1 village leader respondents. However, at several points in the results sections, the writing indicates that there were multiple respondents in these groups. (‘Staff from the local health authority felt…’, and ‘ Village leaders were interested because…’. The text needs to more accurately reflect the singular nature of these responses, and of the other interviewed groups that contained a single respondent. 

    

 I wonder if sustainability is a relevant theme to be discussed this paper, based on the results of the data analysis that are described. The actual responses cited in this section refer to better understanding about malaria elimination projects that were gained through the engagement approach, which would seem to fit under theme 3 (understanding and learning about malaria using drama). Also, increased willingness of villagers to do blood testing does not speak to the sustainability of the engagement approach that is being evaluated, but rather to its impact. I would suggest revisiting this section and thinking about which parts are results that could be included under other themes, and which parts could be moved to the methods or discussion sections.  

     

 The conclusions that are drawn are based on both the rates of participation in each village (averaging out at 66%) and the results from interviews and focus group sessions. In the section on data collection, the authors do acknowledge that their sampling was limited by practicalities, but the small sample size of each respondent group is a key limitation and this needs to be noted in the conclusion section.

**Additional Comments**

 In the results section of the abstract, it is indicated that 'The event saw a very positive response...' Again this indicates that – even although the project included community engagement activities in 20 villages - the article is based on a single engagement event. If this is the case, readers should be made aware of this in the abstract.

 In the conclusion of the abstract, it is stated that 'the drama project was feasible in promoting awareness and understanding of malaria prevention and control'. This statement could be qualified by adding 'in this context' (i.e. rural settings in S.E. Asia). I would make the same suggestion for the final concluding statement in the paper.

 In the discussion, it would have been interesting to hear some of the authors reflections on the ethical considerations of using this specific approach to community engagement, having gained this important experience. For example, as far as I can see, approvals for the use of social media, filming and photography were only gained from the provincial authorities. Is this level of approval sufficient? If so, why is this considered to be the case? Did the project process foster expectations about the elimination of malaria in the district? If so, how were such expectations managed by the MORU team?    

 In the discussion, a sentence or two indicating the authors learning from using a professional, touring drama team as the lead performers in a participatory process would also have valuable.

 Page 3. The explanation of literacy rates needs to be clarified For example : The 2014 census indicated literacy rates to be 93% and 77% in urban and rural areas, respectively. 

 On page 5, the authors explain that approval for the main script, storyline and key messages was sought from provincial health authorities. Was approval for these things also sought, at any stage, from villagers, VMW or local health staff? 

 The authors explain that the average number of attendees at each village performance was 430. This is a large audience size for a community drama event. Were there any implications of this, for example background noise levels, or children running around? Were microphones/speakers used in this process? I wonder if this element of the drama engagement was explored through the interviews or focus groups. These are also important methodological details for the purposes of replicability.     

**Minor comments**

 In five cases, the spelling/formatting of the village names differs between the map (Figure 1) and Table 1.    

 The ethical statement on page 5 is duplicated on page 8.  

 This interesting research article provides important insights into the possibilities and challenges for multi-pronged, creative community engagement strategies to contribute to malaria elimination in rural South East Asia. With a little more reference to directly resonant publications, greater attention to  grammar in some places, the repositioning of certain content, and the inclusion of ethical reflection, it will make an excellent and needed contribution to the community engagement in global health literature.

I have read this submission. I believe that I have an appropriate level of expertise to confirm that it is of an acceptable scientific standard, however I have significant reservations, as outlined above.

## References

[1] FrishkopfMHamzeHAlhassanMZukpeniIAbuSZakusD: Performing arts as a social technology for community health promotion in northern Ghana. *Family Medicine and Community Health*.2016;4(1) : 10.15212/FMCH.2016.0105 22-36 10.15212/FMCH.2016.0105

[2] KingKFKolopackPMerrittMWLaveryJV: Community engagement and the human infrastructure of global health research. *BMC Med Ethics*.2014;15: 10.1186/1472-6939-15-84 84 10.1186/1472-6939-15-84 25495054PMC4290104

[3] WallersteinNDuranB: Community-based participatory research contributions to intervention research: the intersection of science and practice to improve health equity. *Am J Public Health*.2010;100 Suppl 1: 10.2105/AJPH.2009.184036 S40-6 10.2105/AJPH.2009.184036 20147663PMC2837458

[4] MitchellCMSommerM: Participatory visual methodologies in global public health. *Glob Public Health*.11(5-6) : 10.1080/17441692.2016.1170184 521-7 10.1080/17441692.2016.1170184 27105078
